# Exocrine Pancreatic Insufficiency During the Use of Semaglutide: A Case Report

**DOI:** 10.7759/cureus.55549

**Published:** 2024-03-05

**Authors:** Clivaldo Agra de Melo Junior, Norberto Eloi Gomes Júnior, Marco Aurélio da Silva Ribeiro-Sousa, Selma Freire de Carvalho Cunha

**Affiliations:** 1 Division of Clinical Nutrition, Department of Internal Medicine, School of Medicine of Ribeirão Preto, University of São Paulo, Ribeirão Preto, BRA

**Keywords:** obesity, pancreatitis, exogenous pancreatic insufficiency, glp-1 analogue, semaglutide

## Abstract

Although acute pancreatitis has been reported as an adverse event in patients treated with glucagon-like peptide-1 (GLP-1) analogues, to date we have not identified a case describing exocrine pancreatic insufficiency related to this drug. Here we present a case of a patient with no history of acute pancreatitis, who was diagnosed with exocrine pancreatic insufficiency during the third year of weekly subcutaneous semaglutide for obesity control. A 68-year-old man with previous unsuccessful therapeutic approaches for weight loss used subcutaneous semaglutide, once a week, for 181 weeks. The pharmacological treatment, combined with instructions on lifestyle changes, resulted in a weight loss of 11.6% of initial body weight, with a reduction in body mass index from 35.7 to 31.9 kg/m^2^. The patient received a regular dose of 0.5mg/week in the first 14 weeks in a private physician's office. During this period, an asymptomatic elevation of serum lipase was identified and attributed to alcohol consumption. Because the patient complained of less satiety and weight loss stabilization, the dose of semaglutide was increased to 1.0mg/week. Serum lipase levels increased to up to eight times the upper normal limit and the use of semaglutide was discontinued for 10 weeks. Due to progressive weight regain and serum lipase levels being near normal, semaglutide was reintroduced at 0.25mg/week with progressive increments until 1.0mg/week. The patient missed the follow-up appointments, continued the same dose of semaglutide for another 16 weeks, and discontinued treatment after another asymptomatic elevation in serum lipase levels. Four weeks later, the patient reported steatorrhea and was seen by a gastroenterologist, who diagnosed exocrine pancreatic insufficiency. The course of our case suggests a potential association between semaglutide and chronic alcohol consumption in the development of exogenous pancreatic insufficiency. We call attention to the importance of regular monitoring of serum lipase levels in patients taking GLP-1 analogues and suggest that the consumption of any amount of alcohol should be discouraged in patients in long-term treatment with glucagon-like peptide-1 receptor agonists.

## Introduction

Obesity is a chronic recurrent condition with an increasing prevalence that is associated with severe clinical complications and has a negative impact on public health worldwide. Treatment options include lifestyle change, behavioral therapy, bariatric surgery, and a limited number of drugs [1]. Glucagon-like peptide-1 receptor agonists (GLP-1 RAs) are a class of medications primarily developed for the treatment of type 2 diabetes that have shown a favorable effect on cardiovascular risk reduction [2]. Recently, GLP-1 RAs have been increasingly used in the treatment of obesity, even in individuals without diabetes [3]. In June 2021, the Food and Drug Administration (FDA) approved semaglutide, once weekly, as an option for weight loss [4].

Pharmacological safety is a major concern for physicians, especially regarding novel agents for long-term treatment of chronic diseases. Adverse events secondary to the use of GLP-1 RAs such as nausea, vomiting, diarrhea, constipation, and abdominal fullness have been reported in early clinical trials conducted on diabetic patients. The FDA Adverse Event Reporting System (FAERS) has provided updates on GLP-1 RAs’ gastrointestinal safety profiles. Between January 2018 and June 2022, a total of 21,281 reports of gastrointestinal adverse events were identified, and 26.2% of these were for semaglutide. The six most common gastrointestinal events were nausea (42.2%), diarrhea (21.9%), vomiting (21.9%), upper abdominal pain (9.8%), constipation (8.2%), and pancreatitis (8.2%) [5]. The use of any GLP-1 RA was associated with the risk of pancreatitis, with emphasis on the use of liraglutide and semaglutide [5].

However, a meta-analysis of 43 randomized clinical trials showed no clear evidence of risk for pancreatitis, although data on pancreatic cancer were too scarce to conclude [6]. To date, the longest follow-up period (average, 39,8 months) of patients receiving once-weekly semaglutide at a dose of 2.4 mg was the SELECT study, which did not show an increased risk of pancreatitis in obese nondiabetic patients [7]. In this report, we present a case of a man who developed exocrine pancreatic insufficiency at the end of the third year of treatment with subcutaneous semaglutide for weight control.

## Case presentation

The patient was seen in a private office in the city of São Luis, Maranhão, Brazil. All data were obtained from patients’ electronic medical records and all laboratory tests were performed at the same clinical analysis laboratory. The patient signed the informed consent form, and the study was approved by the ethics committee of the General Hospital of Ribeirao Preto Medical School, University of Sao Paulo (approval number 6.540.168).

A 68-year-old man frequently traveled for work and was away from his family for one to two weeks a month. The patient reported a history of being overweight for 30 years and previous unsuccessful therapeutic approaches for weight loss, such as orlistat and sibutramine. He had no personal or family history of type 2 diabetes or dyslipidemia and consumed alcoholic beverages regularly (2-20 doses per week), which represents a risk factor for the development of pancreatitis. The patient had a diagnosis of bipolar affective disorder and was using divalproex sodium (500mg/day). The patient started treatment with semaglutide with a body weight of 112 kg and a body mass index (BMI) of 35.7 kg/m². 

He was instructed regarding lifestyle changes, including a reduction in his usual alcohol consumption, which was 200 grams per week. Concomitantly, subcutaneous semaglutide was started once a week, with an initial dose of 0.25mg, and increased to 0.5mg in the fifth week.

**Figure 1:**
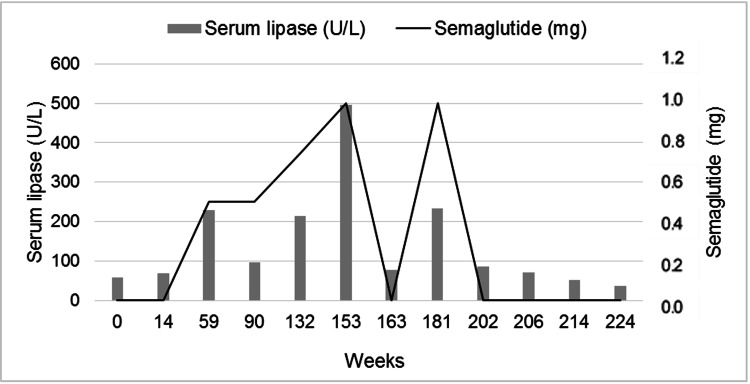
Serum lipase levels and semaglutide doses: according to the weeks of follow-up

The patient had a favorable response to the drug treatment, with a loss of 11.6% of initial weight (Table 1). In the first 14 months of follow-up, the patient attended regularly scheduled visits, without complaints of adverse events other than constipation during the initial weeks of semaglutide. We detected an increase in serum lipase (Figure 1), which exceeded four times the upper normal limit (228 U/L) in the 54th week of treatment. This rise in serum lipase was initially attributed to alcohol abuse, which the patient reported as occasional. He was absent from medical follow-up for 31 weeks, in part because he could obtain the drug without a prescription, and also because laboratory results were interpreted by a physician in the family.

**Table 1:** Dose of semaglutide, body weight, and laboratory data over the treatment period *initiation of bupropion/naltrexone; BMI: body mass index; GGT: gamma-glutamyl transferase; ALT: alanine transferase; hs CRP: high-sensitivity C-reactive protein.

Body weight and laboratory data	Duration (weeks)													
	0	14	59	90	132	153	163		181	202	206*		214	224
Semaglutide (mg/week)	0	0.5	0.5	0.5	0.75	1.0	0		1.0	0	0		0	0
Body weight (kg)	112	104	99	99.9	100	99.9	103.5		-	106	109		-	104
BMI (kg/m^2^)	35.7	33.2	31.9	31.9	31.9	31.9	33		-	33.8	34.8		-	33.2
Serum lipase (< 60U/L)	59	68	228	96	213	495	78		234	85	70		52	37
Amylase (< 100U/L)	-	-	-	-	-	-	45		-	54	45		46	36
Gama GT (< 73U/L)	16	12	11	10	10	11		-	9	11		11	12	9
ALT (<41 U/L)	16	16	18	15	14	15		-	12	15		17	14	13
hs CRP (<1.0 mg/dL)	0.05	0.09	0.04	0.06	0.04	0.06		0.06	0.06	0.05		0.05	0.10	-
Ferritin (< 446 ng/ml)	-	-	349	-	313	280		-	322	262		289	230	-
Fasting glucose (< 100 mg/dL)	89	91	94	87	-	92		-	92	-		-	-	88
Triglycerides (< 150 mg/dL)	150	151	105	114	-	103		-	109	-		-	-	114
Calcium (< 10.3 mg/dL)	-	9.3	-	9.1	-	9.1		-	9.4	9.0		-	-	9.4
25(OH)D (> 30 ng/mL)	-	-	-	48	-	45		-	65	52		-	-	59
Vitamin A (> 0.3 mg/L)	-	-	-	-	-	-		-	-	-		0.7	-	0.7
Zinc (> 0.5 mcg/mL)	-	-	-	-	-	0.81		-	-	-		-	1.0	0.76
Iron (> 65 mcg/dL)	-	-	-	-	-	-		-	-	-		179	59	104
Fecal elastase (>200 µg/g)	-	-	-	-	-	-		-	-	112		151	-	123

During a visit in the 90th week, the dose of semaglutide was gradually increased to 1.0mg per week because the patient complained of less satiety and weight loss stabilization. Other elevations in serum lipase were noted, reaching eight times the upper normal limit (495 U/L) in the 153rd week of treatment. Lipase concentrations decreased (78U/L) 10 weeks after semaglutide was discontinued, which was associated with a reduction in alcohol intake. Due to progressive weight regain, serum lipase levels being near normal, and maintaining no changes in amylase, liver enzymes, and CRP, semaglutide was reintroduced at 0.25mg/week with progressive increments every four weeks until 1.0mg/week. 

Since the patient missed the follow-up appointments and remained without complaints, he continued the same dose of semaglutide for another 16 weeks and discontinued treatment after another asymptomatic elevation in serum lipase levels, which was not reported to the physician.

During the following weeks, the patient had abdominal pain and distension, loose stool, and diarrhea (up to eight evacuations per day). The patient reported steatorrhea in the 180th week of treatment. The patient was seen by a gastroenterologist who diagnosed exocrine pancreatic insufficiency, based on the examination of stool samples for the presence of fat by Sudan III staining (> 200 mcg/g of stool). The patient reported improvement in symptoms with the use of 25,000 IU of pancreatin in the three main meals.

Elevations in serum lipase were asymptomatic and not accompanied by other laboratory changes suggestive of excess alcohol consumption or other markers suggestive of inflammation. There were no alterations in nutritional markers. Measurement of fecal elastase was repeated twice to rule out the possibility of laboratory error, with values remaining low (Table 1).

**Figure 2:**
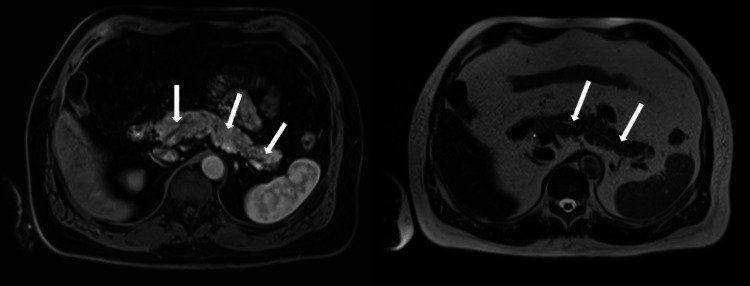
T1-weighted magnetic resonance imaging Pancreatic body and tail without parenchymal changes suggestive of chronic pancreatitis (at left); pancreatic body and tail, and duct without dilatation (at right).

**Figure 3:**
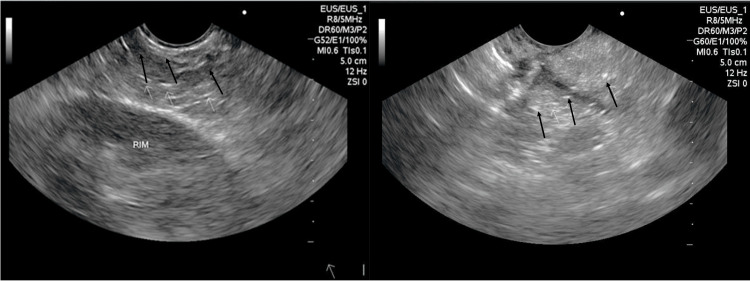
Endoscopic ultrasound Diffuse hyperechoic strands in the parenchyma (arrows) suggestive of initial stage of chronic pancreatitis.

Abdominal magnetic resonance imaging performed at 190 weeks after treatment onset revealed a normal-sized pancreas, without calcifications, pancreatic cyst of 0.6 cm, and hepatic and renal cysts measuring less than 1.4cm (Figure 2) not suggestive of chronic pancreatitis. Endoscopic ultrasound performed at 224 weeks after treatment onset showed heterogeneous parenchyma in the uncinate process and body of the pancreas, with diffuse hyperechoic strands in the body and noncontiguous lobules (Figure 3); morphological changes were suggestive of the initial phase of chronic pancreatitis. 

Since the patient had regained 76% of the weight loss, bupropion was started with increasing doses (90 - 270 mg/day) and naltrexone from 8mg to 24 mg a day at the 206th week of follow-up (Table 1). At 12 weeks after this therapeutic regimen, the patient was taking pancreatin in the three main meals, with pancreatic lipase within the normal range and no gastrointestinal complaints. The nutritional approach included a 1800kcal diet consisting of 34% protein, 44% carbohydrate, 22% lipid, and adequate amounts of micronutrients. The patient was satisfied with the discrete weight loss obtained with the new therapeutic regimen.

## Discussion

During the first year of treatment, the patient had satisfactory weight loss (11.6%) with good adherence to semaglutide (0.5 mg/week) combined with lifestyle change. The weight loss observed in the present case was similar to the average weight loss observed in studies on the efficacy of semaglutide [8]. In studies with nondiabetic obese people, the target dose of semaglutide is 2.4mg weekly [9], which represents almost five times the dose used by our patient.

The patient did not have clinical manifestations compatible with acute pancreatitis during treatment. In an analysis of 5,442 reports of semaglutide-related gastrointestinal adverse events, 389 cases of pancreatitis were identified, with a median time-to-onset of 23 days [10]. The review of 120 cases and case series of GLP-1 RA-associated adverse events published between 2006 and 2021 showed that pancreatitis was reported in 14 patients using liraglutide and in six patients using exenatide, with no reports of chronic pancreatitis or exocrine pancreatic insufficiency [11].

An epidemiological study developed from a large health claims database of outpatient prescriptions and physician diagnoses in the US showed that the use of GLP-1 agonists by nondiabetic obese subjects was associated with a nine times greater risk of pancreatitis as compared with bupropion-naltrexone [12]. Recently, acute pancreatitis was reported in a type 2 diabetic patient after two months of treatment with once-weekly semaglutide 0.5 mg [13]. The authors warned that physicians should be cautious when prescribing this drug in diabetic populations with increased risk factors for acute pancreatitis, such as obesity, prolonged diabetes, and the use of some diuretics, anticonvulsants, and immunosuppressants [13]. After excluding the most common causes of acute pancreatitis, the use of semaglutide for the treatment of type 2 diabetes and SARS-CoV-2 infection was implicated in the development and severity of severe, complicated acute pancreatitis with pancreatic pseudocysts [14]. Although the SUSTAIN-6 trial (2016) showed that diabetic patients using subcutaneous semaglutide did not have an increased risk of developing acute pancreatitis, Patel et al. (2023) suggested that GLP-1 RAs should be discontinued in case of acute pancreatitis, particularly in the presence of other risk factors for the disease [13,15].

Observational studies have reported an increase in serum amylase and lipase in patients using GLP-1 analogues [16]. In the LEADER trial, conducted with 9,000 type 2 diabetic patients, liraglutide was associated with elevations in lipase and amylase, but these laboratory findings were not considered predictive factors of acute pancreatitis [16]. During 56 weeks of treatment of obesity with liraglutide (3 mg/day), the SCALE study did not report the development of acute pancreatitis, although asymptomatic elevations in amylase and lipase were detected, which did not exceed three times the reference values [17].

Approximately 45 months of treatment with semaglutide, our patients had gastrointestinal symptoms characteristic of exocrine pancreatic insufficiency - established by the presence of fecal fat (Sudam III) and abnormal fecal elastase-1 levels (an indirect method to assess exocrine pancreatic insufficiency) - and clinical improvement after pancreatic enzyme replacement therapy. The patient’s clinical data and absence of typical symptoms prior to the third year of treatment with semaglutide indicate that he did not have previous pancreatic insufficiency. However, considering current clinical data, we cannot rule out that the patient had subclinical chronic pancreatitis, not necessarily related to the continuous use of semaglutide.

Exocrine pancreatic insufficiency may be identified within 10 and 12 years after diagnosis of chronic pancreatitis in 60-90% of the cases. Screening for pancreatic insufficiency includes evaluation of gastrointestinal symptoms (e.g. steatorrhea), nutritional blood markers, and abnormal pancreatic tests. The combination of two of these should be considered sufficient for the diagnosis and treatment with pancreatic enzyme replacement therapy [18]. Although our patient had a BMI above normal, the possibility of sarcopenic obesity, which is considered a type of malnutrition, cannot be ruled out.

In our case, the typical findings of chronic pancreatitis, such as pancreatic atrophy, calcifications, calculi, or pseudocysts, and duct dilatation, were detected by neither computed tomography nor magnetic resonance cholangiopancreatography. Endoscopic ultrasonography, which is indicated only when computed tomography and magnetic resonance are inconclusive [19], revealed changes suggestive of pancreatic inflammation, compatible with the initial phase of chronic pancreatitis. Asymptomatic elevations of serum lipase improved with temporary discontinuation of semaglutide; the levels increased again after reintroduction of semaglutide and were maintained within normal ranges after complete discontinuation of this medication and initiation of other therapeutic scheme for obesity. We cannot exclude the possibility that exocrine pancreatic insufficiency may have been caused by autoimmune pancreatitis, genetic polymorphism, or medications. This single case suggests that semaglutide may be associated with the development of pancreatic insufficiency in patients with possible chronic pancreatitis related to chronic alcohol abuse.

## Conclusions

Considering that alcohol consumption is the main cause of chronic pancreatitis, we cannot attribute the cause of exocrine pancreatic insufficiency solely to semaglutide. However, the course of our patient suggests an association between chronic use of semaglutide for obesity and alcohol abuse in the development of exocrine pancreatic insufficiency. There are few studies on the use of semaglutide for the treatment of obesity for long periods, and hence the chronic effects of this drug on exocrine pancreatic function still need to be clarified. We call attention to the importance of regular monitoring of serum lipase levels in obese patients taking GLP-1 analogues and the assessment of clinical manifestations of pancreatic insufficiency. Although we did not find any study on this issue, we suggest that the consumption of any amount of alcohol should be discouraged in patients in long-term treatment with GLP-1 RAs.
